# Model-informed prediction of antiviral drug combination synergy from sparse data using complementary mechanistic and pharmacodynamic approaches

**DOI:** 10.3389/fphar.2026.1834793

**Published:** 2026-07-03

**Authors:** Yongdae Jeong, Jong Hyuk Byun

**Affiliations:** 1 Department of Mathematics and Institute of Mathematical Sciences, College of Natural Sciences, Pusan National University, Busan, Republic of Korea; 2 Institute for Future Earth, Pusan National University, Busan, Republic of Korea

**Keywords:** antiviral combinations, bliss pharmacodynamic model, mechanistic modeling, synergy prediction, viral dynamics

## Abstract

**Introduction:**

Optimization of antiviral drug combinations remains challenging because exhaustive measurement of dose–response spaces is experimentally impractical. Although Bliss-based pharmacodynamic models are commonly used to describe combination responses, their empirical nature limits biological interpretation.

**Methods:**

To address these limitations, we developed a model-informed framework to reconstruct antiviral combination response surfaces from sparse diagonal observations by combining a Bliss-based pharmacodynamic model with a mechanistic viral dynamics model.

**Results:**

Both models inferred unmeasured response regions and quantified synergy relative to Bliss independence through an interaction parameter. The mechanistic viral dynamics model reconstructed response surfaces more accurately than the Bliss-based model while retaining a biologically interpretable structure linked to viral infectivity and production.

**Conclusion:**

These results provide a practical modeling strategy for antiviral combination optimization under limited-data experimental settings.

## 1 Introduction

Combination drug therapies play a pivotal role in combating infectious diseases by enhancing efficacy, mitigating resistance, and targeting multiple pathways (Khan et al., 2019; Mangel et al., 2001). For example, Lamivudine plus interferon shows improved viral suppression in HBV compared with monotherapy (Korba et al., 2000; Colombatto et al., 2006), interferon with PGA1 controls HTLV-I infections effectively (D'Onofrio et al., 1993), and remdesivir with interferons accelerates viral clearance in SARS-CoV-2 (Gastine et al., 2021). Despite these successes, selecting optimal combinations remains challenging because experimental dose–response matrices grow rapidly with the number of drugs and concentrations, making comprehensive screening expensive and time-consuming. In many practical settings, only sparse measurements are available, and a central question is whether such limited observations can reliably support prediction of efficacy and synergy in untested regions.

Most predictive approaches for drug combinations still rely on empirical statistical models, including Bliss independence and Loewe additivity (Chou, 2006; Chou, 2010). Bliss independence assumes independent drug action and computes the expected combination effect from the product of unaffected fractions; synergy is then quantified as the deviation between the observed efficacy and this expectation. While useful and widely adopted, Bliss independence does not explicitly incorporate biological mechanisms such as viral dynamics or PK/PD processes. The Bliss pharmacodynamic (PD) extension partially addresses nonlinearity by introducing an empirical interaction parameter ( α ) that allows deviation from the Bliss-based response (Ianevski et al., 2019; Rosario et al., 2006). However, even with improved flexibility, the Bliss-PD formulation remains phenomenological and offers limited mechanistic interpretability for why synergy changes across dose regions (Yoshiki Koizumi and Iwami, 2014). Data-driven machine-learning approaches can improve predictive accuracy for certain antiviral combinations (e.g., acyclovir and Ribavirin), yet they often suffer from limited interpretability and may not capture biologically meaningful interaction structures, especially when data are sparse or extrapolation is required (Shayan Majidifar et al., 2024; Vakil and Trappe, 2019).

Mechanistic mathematical models provide a biologically grounded alternative by directly embedding parameters such as infectivity and viral production into therapy evaluation (Fitzgerald et al., 2006). Such models can support rational design of antiviral combinations, but their use for synergy prediction under sparse experimental designs remains underexplored, particularly in emerging infections such as SARS-CoV-2. Prior studies of host- and virus-targeting combinations report strong inhibitory effects but also highlight the need to connect synergy patterns to mechanisms (Wagoner et al., 2022). Likewise, combinations targeting entry pathways such as TMPRSS2 and Cathepsin B/L emphasize the importance of mechanistic insight for optimizing therapy across dose ranges (Padmanabhan et al., 2020). In this context, we sought to determine whether sparse combination measurements could support efficacy prediction and synergy assessment in untested regions. While empirical benchmark models such as Bliss-PD are useful for describing combination effects, they provide limited mechanistic interpretability. We therefore formulated a viral dynamics model in which drug actions directly modulate key biological parameters (e.g., infectivity and viral production), enabling a more biologically grounded description of combination effects.

**FIGURE 1:**
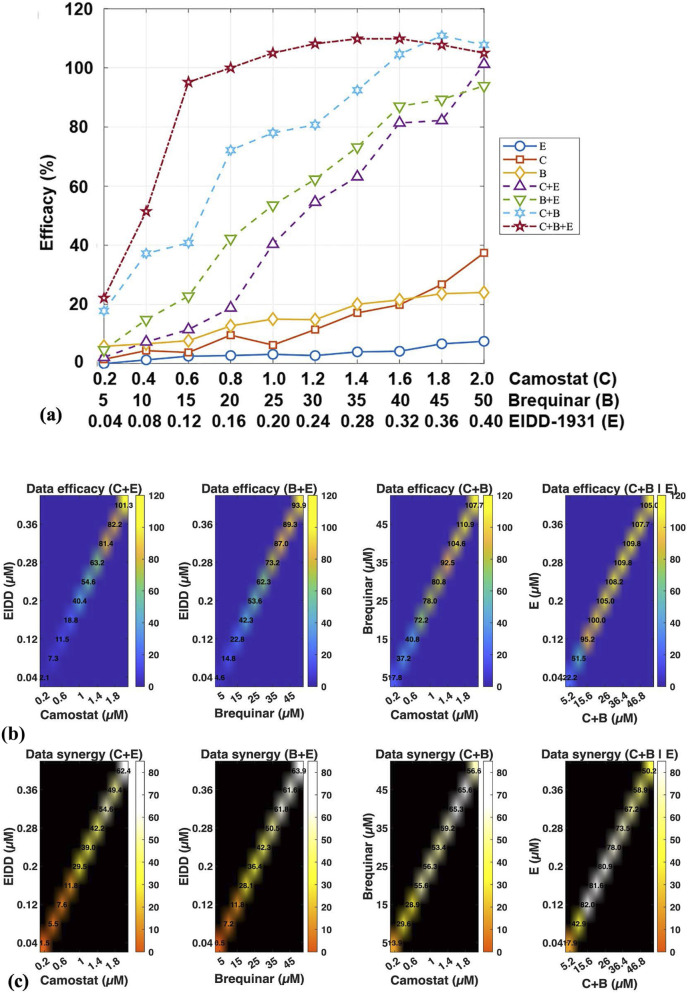
Observed efficacy (data). **(a)** Three drugs, Camostat ( C ), Brequinar ( B ), and EIDD ( E ), together with combination efficacies were measured at specific concentrations ( µM ). **(b)** From (a), Efficacies were performed only on the diagonal dose pairs (ten dose points), shown as heatmaps. Unmeasured efficacies left blank. **(c)** Synergy scores computed under Bliss independence, defined as $S=E_{obs}-E_{combo}$ . For the triple combination (C+B+E), the diagonal slice was visualized by matching the same dose level across all three drugs, with the x-axis representing the summed C and B doses and the y-axis representing the corresponding E dose.

**FIGURE 2:**
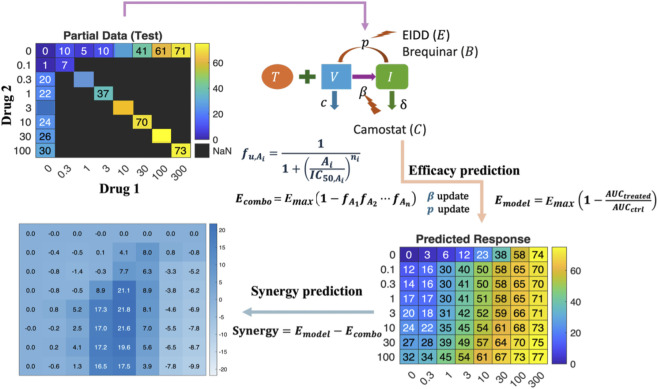
Study workflow for predicting efficacy and quantifying synergy from partial combination data. A partially observed dose–response matrix (test setting; unmeasured entries shown as NaN) is used for model fitting. A mechanistic viral model incorporates drug actions by modulating infection rate β (Camostat) and viral production rate p (Brequinar and/or EIDD), with clearance and death terms ( c,δ ). Drug effects are parameterized by Hill unaffected fractions $f_{u,A_{i}},$ yielding the modeled efficacy $E_{model}=E_{\max}\left(1-\text{AU}C_{treated}/\text{AU}C_{ctrl}\right).$ The fitted model predicts efficacy in unobserved regions (predicted response heatmap). Synergy scores are quantified relative to Bliss independence as the deviation between the model-predicted efficacy and the Bliss-expected efficacy, $S=E_{model}-E_{bliss}$ , and visualized as a synergy heatmap.

In this study, we develop a data-efficient framework to predict untested efficacy landscapes and quantify synergy from partially observed combination data by integrating two complementary models: a Bliss-PD independence model (benchmark) and a mechanistic viral model. A schematic overview of this workflow is provided in Figure 2, where partial diagonal observations are used for model fitting, followed by full-grid efficacy reconstruction and Bliss-referenced synergy quantification. Specifically, we study three antivirals—Camostat (C), Brequinar (B), and EIDD-1931 (E)—and their combinations. To address realistic data limitations, Using experimentally measured efficacy data for Camostat, Brequinar, and EIDD-1931 and their pairwise/triple combinations (Figure 1) (Wagoner et al., 2022), we emulate a realistic sparse setting by retaining only a subset of diagonal measurements for model fitting. The fitted models then extrapolate to the full concentration grid to reconstruct efficacy heatmaps and compute synergy as the deviation from Bliss independence, including a diagonal projection for the triple-combination visualization. Across combinations, both models generate coherent efficacy landscapes, while their implied synergy structures differ in a biologically informative way: synergy generally increases with dose yet can attenuate at very high concentrations, and this attenuation at extreme doses is most clearly expressed by the viral model, particularly in the C+B and projected triple (C+B | E) matrices. Quantitatively, observed-versus-predicted synergy comparisons (Figure 6) show that the viral model yields improved agreement with measured synergy (lower error) relative to the Bliss-PD benchmark. Together, these results indicate that reliable synergy prediction is feasible even under sparse experimental designs, and that incorporating mechanistic viral dynamics can improve both predictive performance and interpretability. This comparative framework strengthens evaluation of antiviral drug combinations and supports practical prioritization and optimization of combination therapies under realistic data constraints (Meng et al., 2016; Finch et al., 2021; Gautam et al., 2016).

## 2 Methods

### 2.1 Data source and study design

**FIGURE 3:**
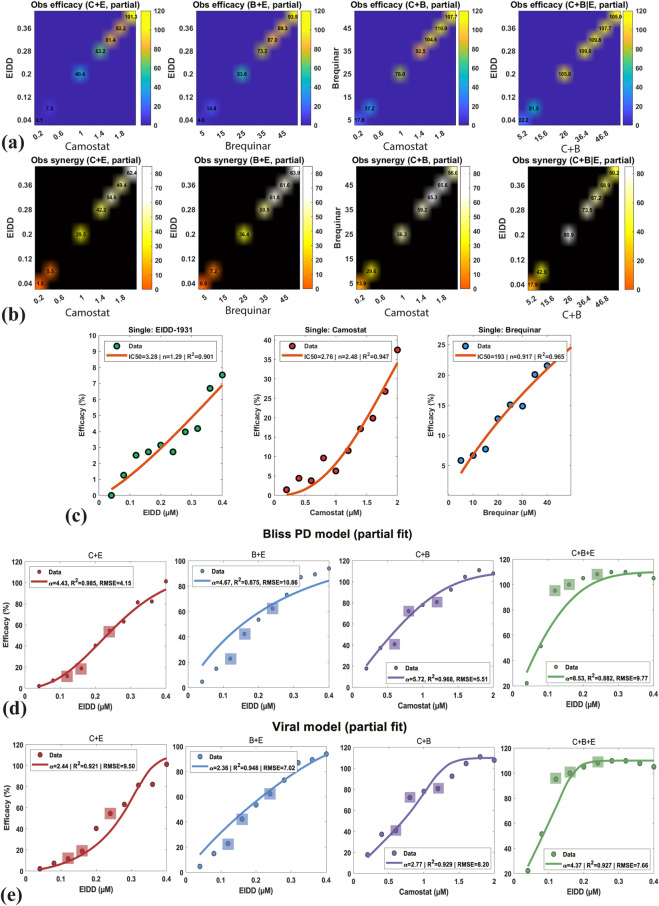
Partial-data setting and fitting accuracy for single and combination treatment. **(a)** Observed combination efficacies shown as heatmaps under a diagonal-only experimental design; only 7 of 10 diagonal measurements were used for model fitting, and the remaining entries are unobserved. **(b)** Corresponding observed synergy on the same partial grid, computed as (Observed efficacy − Bliss-independence expectation); boxed entries indicate held-out test points not used for parameter estimation. **(c)** Single-drug dose–response curves fitted with a (sigmoid) Emax/Hill model, reporting goodness-of-fit (*R* ^2^). **(d)** Combination responses along the diagonal (matched dose indices) fitted using the Bliss PD model (benchmark). **(e)** The same diagonal combination responses fitted using the viral model. Across combinations, the viral model provides comparable or improved fit quality, with clearer gains for B+E and C+B+E (higher *R* ^2^ at the diagonal observations), supporting its use for predicting unobserved efficacy and downstream synergy evaluation.

This study was designed and implemented based on the antiviral dose–response and combination dataset reported by Wagoner et al. (Figure 1) (Wagoner et al., 2022). The source dataset was generated from *in vitro* live-virus SARS-CoV-2 experiments, primarily in Calu-3 human lung epithelial cells and, in selected analyses, 293TAT cells expressing ACE2 and TMPRSS2. In those experiments, cells were pretreated with antiviral compounds and then infected with wild-type SARS-CoV-2 WA1 or selected variants, and the primary assay readout was CellTiter-Glo-based cell viability/luminescence, from which antiviral efficacy and dose–response measurements were derived. To mimic a sparse experimental setting, we constructed a diagonal-only, partial-observation design in which only seven of 10 diagonal measurements per combination were used for model fitting and the remaining three were held out for evaluation (Figures 3a,b). Using these measurements as ground truth, we benchmarked two predictive frameworks—the Bliss pharmacodynamic (Bliss PD) model and a mechanistic viral dynamics model.

### 2.2 Computational pipeline overview

We first fitted the single-drug dose–response curves for EIDD-1931, Camostat, and Brequinar to obtain the $IC_{50}$ and Hill coefficient for each drug. These single-drug fits were then used in both models to define how each drug acts at a given concentration. For each combination (C+E, B+E, C+B, and C+B+E), we used only the diagonal measurements; under the sparse-data setting, seven of the 10 diagonal points were used for fitting and the remaining three were used for evaluation. In the Bliss PD model, the interaction parameter α was fitted to the observed combination efficacy while the single-drug parameters were kept fixed. In the viral model, the same single-drug dose–response functions were used to modify viral infectivity and viral production in the ODE system, α was fitted to the observed combination efficacy, and the kinetic parameters β,p,δ, and c were kept fixed. The ODE system was then solved over time, and the simulated viral-load reduction was converted to efficacy using the treated-versus-control AUC ratio. Finally, the fitted models were used to reconstruct efficacy, calculate Bliss-referenced synergy, and compare predictions with the observed data using root mean squared error (RMSE) and mean absolute error (MAE).

### 2.3 Single-treatment data and single-drug dose–response fitting

Single-drug efficacy measurements for Camostat (C), Brequinar (B), and EIDD-1931 (E) were taken from Wagoner et al. (2022) (Figure 1). These data were used to parameterize the dose–response functions required by both the Bliss PD model and the viral dynamics model.

Antiviral efficacy was computed in the source study from CellTiter-Glo luminescence readouts as:

$$\text{Efficacy}=\left(\frac{IFX_{drug}-avg.IFX_{DMSO}}{avg.non\_IFX_{DMSO}-avg.IFX_{DMSO}}\right)\cdot 100$$

where $IFX_{drug}$ denotes luminescence of infected cells treated with the drug, $avg.IFX_{DMSO}$ is the mean infected DMSO control, and $avg.\;non-IFX_{DMSO}$ is the mean uninfected DMSO control. Because efficacy was defined from a normalized CellTiter-Glo viability readout, values slightly above 100% could occur when the treated infected signal exceeded the average uninfected control signal.

To parameterize the dose–response relationships used in both the Bliss PD and viral models, single-drug efficacy curves were fitted using a sigmoid Emax/Hill function:

$$E\left(D\right)=\frac{E_{\max}D^{n}}{IC_{50}^{n}+D^{n}}$$

where $E\left(D\right)$ denotes the efficacy at drug concentration D, $E_{\max}$ represents the maximal efficacy, $IC_{50}$ is the concentration producing 50% of $E_{\max}$ , and n is the Hill coefficient. In the fitting procedure, $E_{\max}$ was set to 110 to accommodate observed efficacy values slightly above 100% without artificial truncation. Parameters were estimated by nonlinear least-squares regression in MATLAB (MathWorks), and goodness-of-fit was summarized using *R* ^2^.

### 2.4 Combination treatment assay

#### 2.4.1 Combination efficacy data and observed synergy (bliss independence)

Combination efficacy measurements for two-drug (C+E, B+E, C+B) and triple-drug (C+B+E) treatments were obtained from the same experimental setting as the single-drug assays and organized on a dose–response matrix. To emulate a sparse experimental design, only diagonal entries were treated as observed, and a subset of these diagonal points was used for model fitting, with the remaining diagonal points held out for testing (Figures 3a,b). Observed synergy was quantified using Bliss independence as

$$S_{obs}=E_{obs}-E_{combo}$$

where $E_{obs}$ is the measured combination efficacy and the Bliss-expected efficacy is

$$E_{combo}=E_{\max}\cdot \left(1-\Pi_{D\in \left\{C,B,E\right\}}f_{u,D}\right)\;.$$

The unaffected fraction for each drug *D* at concentration *D* was defined as

$$f_{u,D}=1-\frac{D^{n}}{IC_{50,D}^{n}+D^{n}}$$

using ( $IC_{50,D},n_{D}$ ) estimated from the single-drug fits (Figure 3c). Positive $S_{obs}$ indicates synergy and negative values indicate antagonism (Zhao et al., 2014).

#### 2.4.2 Bliss PD model for efficacy prediction and synergy scoring (benchmark)

To predict unobserved efficacies from partial combination data, we used the Bliss pharmacodynamic (Bliss PD) model, which introduces an empirical interaction parameter α to allow deviations from Bliss-based reference response (Finch et al., 2021; Jilek et al., 2012), as shown in Figure 3d:

$$E_{bliss}=E_{\max}\cdot \left(1-{\left(\Pi_{D\in \left\{C,B,E\right\}}f_{u,D}\right)}^{\alpha }\right)$$

In this formulation, α controls the degree of nonlinear deviation from the Bliss-based baseline: α=1 corresponds to the reference case, whereas α>1 and α<1 indicate synergistic and antagonistic deviations, respectively. Single-drug parameters ( ${IC}_{50,D},n_{D}$ ) were fixed from Section 2.3, and α was estimated from the partial (diagonal-only) combination observations by nonlinear least-squares optimization (MATLAB 2025b). Bliss PD synergy was then reported on the same Bliss baseline as

$$S_{bliss}=E_{bliss}-E_{combo}$$

This formulation yields full-grid efficacy and synergy heatmaps by extrapolating from the partial observations.

#### 2.4.3 Viral dynamics model for predicting efficacy and quantifying synergy

To mechanistically predict efficacy at untested concentrations and quantify synergy from sparse combination data (Figure 2), we employed a standard target cell–infected cell–virus (*T–I–V*) model describing the dynamics of target cells *T*, infected cells *I*, and viral load *V* (Wang et al., 2020):

$$dT/\text{dt}=-\beta_{eff}\;T\cdot \;V,dI/\text{dt}=\beta_{eff}T\cdot V-\delta I,dV/\text{dt}=p_{eff}I-cV,$$

Here, $\beta_{eff}$ is the effective infection rate, $p_{eff}$ is the effective viral production rate, δ is the infected-cell death rate, and c is the viral clearance rate. Baseline parameters ( β,p,δ,c ) were taken from a prior study (Wang et al., 2020), and the initial conditions were set to $T_{0}=50,000,I_{0}=0$ , and $V_{0}=5,000$ (MOI = 0.1), consistent with the experimental setting (Table 1).

**TABLE 1:** Fitted parameters in models. This table presents combinations (C+E, B+E, C+B, C+B+E) based on models. Parameter α reflects nonlinearity in drug interactions: α=1 indicates additive effects, α>1 indicates synergy. Only α was estimated from combination data.

Drugs	α (Bliss PD)	α (Viral model)	β	p	δ	c
C+E	4.36 [3.99, 4.96]	2.43 [2.03, 3.38]	2e-7/24	5e3/24 (fix)	0.5/24 (fix)	5/24 (fix)
B+E	4.72 [3.83, 5.48]	2.36 [2.16, 2.47]				
C+B	5.72 [5.36, 6.29]	2.77 [2.17, 3.62]				
C+B+E	8.31 [6.63, 12.58]	4.37 [3.73, 4.64]				

Drug effects were incorporated by modulating biologically meaningful parameters following the reported mechanisms (Wagoner et al., 2022). Camostat (C*)* was modeled to reduce infectivity by decreasing β , whereas Brequinar (B) and EIDD-1931 (E) were modeled to suppress viral production by decreasing p . Let the unaffected fraction for drug D∈C,B,E be

$$f_{u,D}\left(D\right)=1-\frac{D^{n_{D}}}{IC_{50,D}^{n_{D}}+D^{n_{D}}},$$

with $\left({IC}_{50,D},n_{D}\right)$ obtained from single-drug fits.

To capture interaction-related modulation of these drug effects, we introduced an interaction parameter α and defined

$$\beta_{eff}=\beta \cdot {\left(f_{u,C}\right)}^{\alpha },p_{eff}=p\cdot {\left(f_{u,B}\cdot f_{u,E}\right)}^{\alpha }\;.$$

Here, α controls how strongly the single-drug unaffected fractions act on the biologically defined processes in the model.

Model-predicted efficacy was computed from the reduction in viral burden relative to the untreated control using the viral-load area under the curve (AUC):

$$E_{viral}=E_{\max}\cdot \left(1-\frac{AUC_{treated}}{AUC_{ctrl}}\right),$$

where $\text{AU}C_{treated}$ and $\text{AU}C_{ctrl}$ denote the time-integrated viral load under treatment and control conditions, respectively. The parameter α was estimated by fitting $E_{viral}$ to the observed combination efficacy using nonlinear least squares (lsqnonlin) shown in Table 1. The fitted viral model was then used to extrapolate efficacy over the full dose grid (Figure 3e). Synergy score of the viral model was then quantified by comparing the viral model predictions with Bliss independence expectations:

$$S_{viral}=E_{viral}-E_{combo}$$

## 3 Results

To evaluate whether sparse measurements can support reliable synergy prediction, we analyzed a diagonal-only combination design in which efficacy and Bliss synergy were observed at ten matched dose indices, leaving most of the 2D dose matrix unmeasured (Figure 1). We fit both models to partial diagonal observations and reconstructed full-grid efficacy, from which synergy, given by $S_{model}=E_{model}-E_{combo}$ , was quantified as the deviation from Bliss independence. This design enables a direct benchmark of full-surface reconstruction and synergy accuracy under identical sparsity.

### 3.1 Partial diagonal fitting supports identifiability and provides parameters for full-matrix prediction

**FIGURE 4:**
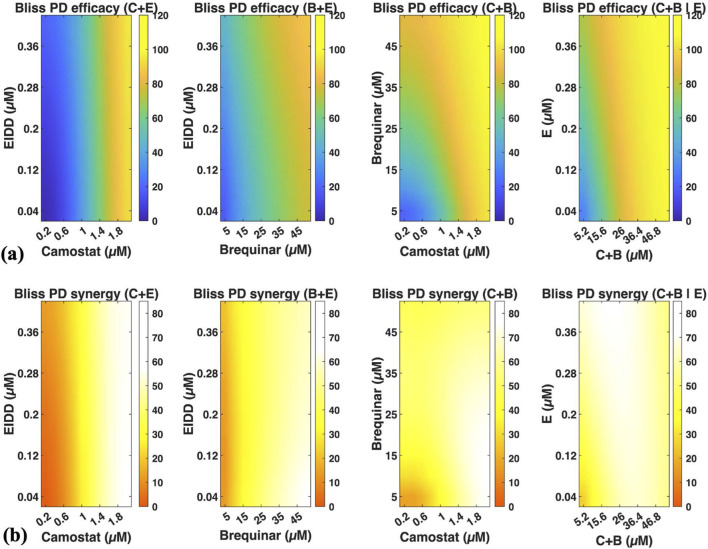
Bliss PD model–based prediction of efficacy and synergy across the dose matrix (benchmark). **(a)** Predicted efficacy (%) for each combination using the fitted Bliss PD model. Heatmaps are shown for C+E, B+E, C+B, and the triple combination are presented as a diagonal projection (C+B | E), where the *x*-axis is the summed concentration $C_{i}+B_{i}$ (matched index pairs) and the *y*-axis is E. Across all panels, efficacy increases with increasing doses, with the triple projection showing the highest predicted responses over most of the grid. **(b)** Predicted synergy computed as the deviation from Bliss independence (i.e., Bliss independence model-predicted efficacy ( $E_{bliss}$ ) minus the Bliss expectation ( $E_{combo}$ ) from single-drug fits). Synergy generally increases with dose, but at very high concentrations it attenuates (decreases), and this high-dose reduction is most evident in the triple-treatment projection (C+B | E).

**FIGURE 5:**
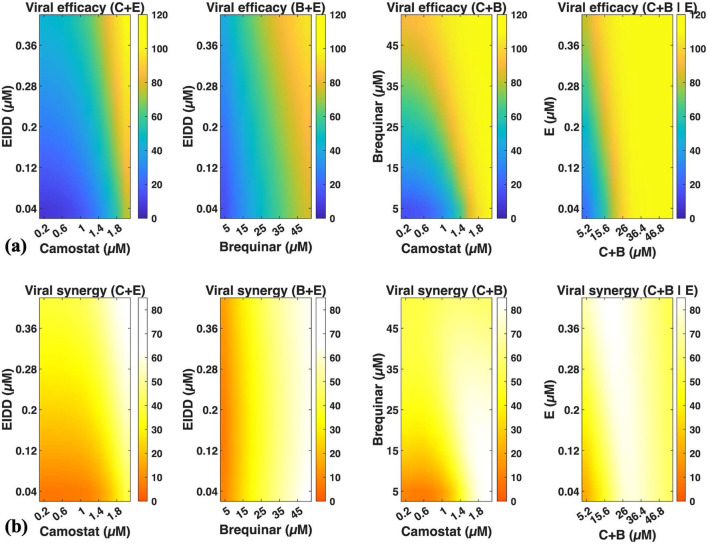
Viral model–based prediction of efficacy and synergy. **(a)** Predicted efficacy matrix for pairwise (C+E, B+E, C+B) and projected triple (C+B | E) combinations obtained from the viral dynamics model fitted to partial observations. Across all combinations, efficacy increases with dose, and the projected triple combination achieves the highest predicted efficacy over most of the concentration range. **(b)** Predicted synergy (defined as $E_{viral}-E_{combo}$ ) computed from the viral-model predictions. Synergy generally strengthens as concentrations increase, but at excessively high concentrations the synergy tends to diminish, most clearly in the C+B and projected triple (C+B | E) panels. Unlike benchmark (Figure 4), the viral model suggests dose-amplified synergy followed by attenuation at extreme doses, most evident for C+B and C+B+E.

**FIGURE 6:**
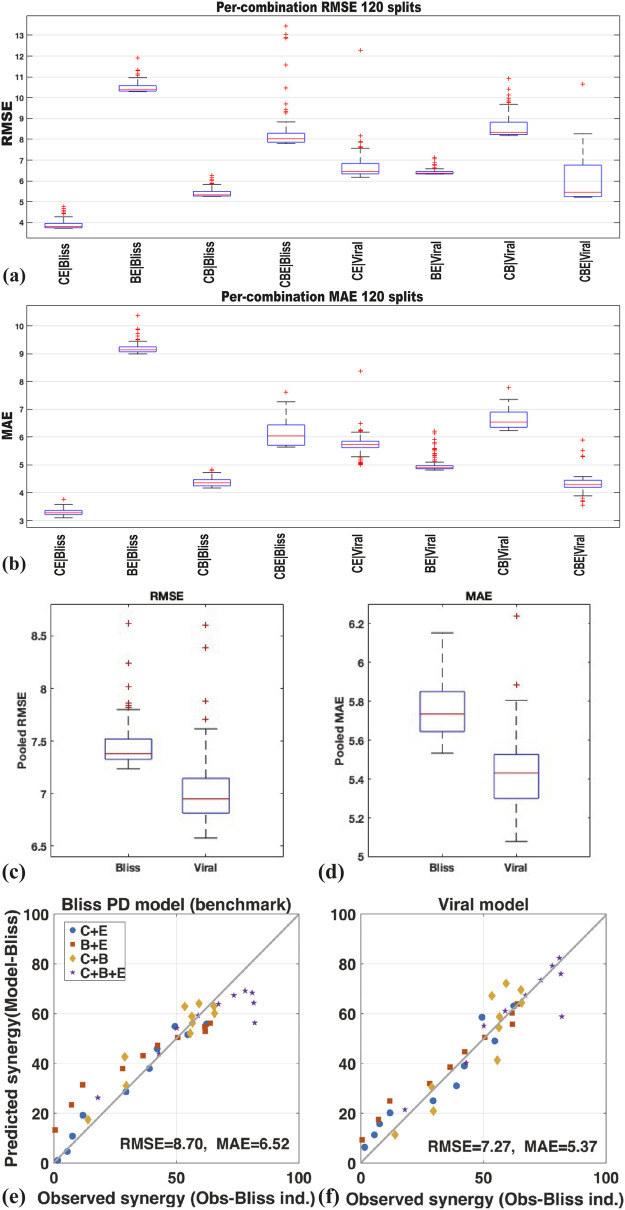
Combination-level and pooled reconstruction accuracy across sparse diagonal partitions. For each of the 120 possible diagonal partitions, α was refitted using seven diagonal samples for model fitting, and prediction errors were evaluated after reconstruction. Per-combination full-diagonal reconstruction errors are shown as RMSE **(a)** and MAE **(b)**. Pooled errors across all 120 partitions are summarized as RMSE **(c)** and MAE **(d)**. Representative observed-versus-predicted synergy comparisons are shown for the Bliss PD benchmark **(e)** and the viral model **(f)**. The *x*-axis shows the observed synergy (Obs ( $E_{data}$ ) −Bliss ind. ( $E_{combo}$ )), and the *y*-axis shows the model-predicted synergy (Model ( $E_{bliss}$ or $E_{viral}$ )− Bliss ind.); the gray line indicates perfect agreement (*y = x*). For the representative partition, the Bliss PD benchmark yielded RMSE = 8.70 and MAE = 6.52, whereas the viral model yielded RMSE = 7.27 and MAE = 5.37.

We tested whether the diagonal-only measurements are sufficient to fit each model reliably. Under the partial-data setting, only seven of 10 diagonal observations were used for parameter estimation, and the remaining points were held out (Figures 3a,b). Single-drug dose–response curves were well captured by the sigmoid Emax/Hill model with high goodness-of-fit (*R* ^*2*^ shown in each panel), providing stable single-drug parameters for computing the Bliss expectation $E_{combo}$ used throughout the study (Figure 3c). The fitted single-drug $IC_{50}$ and Hill coefficient values with 95% confidence intervals are summarized in Table 2. Across combinations, fitted curves closely tracked the observed diagonal trends, supporting parameter identifiability even when trained on seven of 10 diagonal points. Using the same partial diagonal data, both models reproduced the observed diagonal combination responses: the Bliss PD benchmark captured the measured trends across C+E, B+E, C+B, and the triple projection (Figure 3d), while the mechanistic viral model achieved comparable or improved agreement, with clearer gains for B+E and the triple projection (Figure 3e). These partial-data fits establish that key parameters remain identifiable under sparse sampling and provide the fitted model parameters needed for the next step—predicting full efficacy surfaces (Figures 4, 5) and evaluating synergy prediction accuracy against observations (Figure 6).

**TABLE 2:** Single-drug fitted parameters from the sigmoid Emax/Hill model. The table reports ${IC}_{50}$ and Hill coefficient ( n ) estimates with 95% confidence intervals for EIDD-1931, Camostat, and Brequinar.

Drugs	${IC}_{50}$	95% CI	Hill coefficient ( n )	95% CI
EIDD	3.2756	[1.5682, 21.6975]	1.2862	[0.7709, 1.9048]
Camostat	2.7579	[2.5014, 5.0194]	2.4835	[1.3702, 3.1438]
Brequinar	193.3473	[148.80, 273.29]	0.9165	[0.7636, 1.0785]

### 3.2 Full efficacy and synergy surfaces reconstructed from partial diagonal fits (benchmark vs. viral)

Using the parameters estimated from partial diagonal observations (Figure 3), we reconstructed full dose–dose efficacy landscapes over the unmeasured regions for all pairwise combinations (C+E, B+E, C+B) and for the projected triple slice (C+B | E) (Figures 4a, 5a). In both modeling frameworks, predicted efficacy increases monotonically with dose across panels, and the projected triple combination exhibits the highest efficacy over most of the concentration range, consistent with the expectation that targeting complementary mechanisms yields stronger suppression.

Despite similar efficacy landscapes, the synergy surfaces derived from each model show informative structure (Figures 4b, 5b). Under the Bliss PD benchmark, synergy (defined relative to Bliss independence) generally strengthens with dose but shows attenuation at very high concentrations, most prominently in the projected triple (C+B | E) panel (Figure 4b). The viral model yields a qualitatively similar dose-amplified synergy pattern, yet the high-dose attenuation is more pronounced, particularly for C+B and the projected triple combination (Figure 5b). Together, these reconstructed heatmaps demonstrate that sparse diagonal training data are sufficient to generate coherent full-grid efficacy predictions, while the inferred synergy landscapes differ across models in a way that highlights model-dependent differences in the inferred interaction patterns, especially at the high-dose regime.

### 3.3 Quantitative synergy accuracy and inferred interaction strength

Table 1 lists the fitted interaction parameter α used to generate the full efficacy surfaces in Figures 4, 5; these α estimates, together with the fixed viral parameters and single-drug Hill fits, determine how unobserved entries in the dose–response matrices are filled by each model (Figure 6; Table 1).

To compare the model outputs with the observed data on the Bliss-referenced scale, we evaluated observed synergy (computed from data as $S_{obs}=E_{obs}-E_{combo}$ ) against model-predicted synergy (computed as $S_{model}=E_{model}-E_{combo}$ ) across combinations (Figure 6). We performed an exhaustive sparse-data reconstruction analysis over all 120 possible diagonal partitions, each consisting of seven diagonal samples used for model fitting and three held-out diagonal samples used for reconstruction evaluation. At the combination level, this analysis showed heterogeneous model performance: the Bliss PD benchmark showed lower RMSE/MAE for C+E and C+B, whereas the viral model showed lower RMSE/MAE for B+E and C+B+E (Figures 6a,b). Thus, the viral model was not uniformly superior for every individual combination, but its advantage became apparent in the aggregate comparison across combinations and partitions. For the split-based full-diagonal reconstruction analysis, we summarized model performance across all 120 aligned diagonal partitions. In each partition, α was fitted using seven of the 10 diagonal measurements, and reconstruction error was then calculated over the full set of 10 diagonal measurements. The viral model showed lower pooled RMSE than the Bliss PD model in 114 of 120 partitions and lower pooled MAE in 118 of 120 partitions (Figures 6c,d). Mean pooled RMSE and MAE were also lower for the viral model than for the Bliss PD model (RMSE: 7.018 vs. 7.527; MAE: 5.423 vs. 5.754). A representative partition is shown in Figures 6e,f, where observed and model-predicted Bliss-referenced values are plotted across the diagonal points, with pooled RMSE and MAE calculated over the displayed points.

Table 1 summarizes the fitted interaction nonlinearity parameter α for each combination in both frameworks. While α serves as a nonlinear interaction strength parameter in both models, its role differs: in the Bliss PD benchmark it directly shapes the fractional-power interaction in efficacy, whereas in the viral model it modulates biologically meaningful processes (e.g., infectivity β and production p ). Notably, the viral model yields systematically smaller α values than the Bliss PD benchmark across combinations, consistent with the idea that mechanistic propagation in the viral model can reproduce observed interaction patterns without requiring large phenomenological nonlinearity. Overall, the combination of lower RMSE/MAE (Figure 6) and more mechanistically grounded interpretation of α (Table 1) supports the viral model as a stronger framework for synergy prediction under sparse observation settings.

## 4 Discussion

This study demonstrates that antiviral efficacy and synergy can be reconstructed from sparse, diagonal-only combination measurements by integrating two complementary modeling frameworks: a Bliss pharmacodynamic (Bliss PD) benchmark and a mechanistic viral dynamics model. Using the dataset of Camostat (C), Brequinar (B), and EIDD-1931 (E) reported by Wagoner et al. (Wagoner et al., 2022), we trained each model on partial diagonal observations and then extrapolated to the full dose–dose grids to generate efficacy landscapes and corresponding synergy surfaces. Importantly, synergy was quantified consistently as the deviation from Bliss independence, $S_{model}=E_{model}-E_{combo}$ , enabling a direct head-to-head comparison under identical sparsity (Figures 1, 2).

A key finding is that partial diagonal fitting was sufficient for stable parameter estimation and accurate reproduction of observed diagonal trends (Figure 3). Single-drug responses were well captured by Emax/Hill fits (Figure 3c), providing a consistent baseline for computing the Bliss expectation $E_{combo}$ . Using only seven of 10 diagonal measurements for each combination (Figures 3a,b), both models reproduced diagonal combination responses (Figures 3d,e), supporting parameter identifiability even under reduced observations.

When extrapolated to unmeasured regions, both frameworks produced coherent full-grid efficacy landscapes (Figures 4a, 5a), with efficacy increasing with dose and the projected triple slice ( C+B|E ) showing the highest predicted efficacy overall. However, the inferred synergy surfaces revealed model-dependent structure (Figures 4b, 5b): synergy generally increased with concentration but attenuated at extreme doses, with the attenuation most evident in the triple projection and more pronounced in the viral model for C+B and ( C+B|E ). This distinction is biologically relevant because the viral model embeds drug actions into infection and production processes, whereas the Bliss PD model captures interactions phenomenologically through a fractional-power form.

The Bliss-referenced pooled error comparison indicates that both modeling approaches can reconstruct response patterns from sparse diagonal observations. However, across the exhaustive 120-split analysis, the viral model showed a more consistent reduction in pooled prediction error than the Bliss PD benchmark, supporting the practical utility of the viral-dynamics framework under sparse-data conditions.

The Bliss PD model advances beyond traditional Bliss independence by introducing the nonlinear interaction parameter (*α*), which quantifies synergy as a continuous score rather than a binary outcome. This innovation enables a more refined interpretation of drug interactions while preserving computational simplicity. In contrast, the viral dynamics model incorporates drug actions into biologically meaningful parameters, specifically infectivity (*β*) and viral production (*p*), thereby offering mechanistic interpretability that the Bliss framework lacks.

Table 1 summarizes the fitted interaction parameter α , which is a key determinant of how each model fills unobserved entries in the dose–response matrices (Figures 4, 5). In the Bliss PD benchmark, α directly governs the nonlinear fractional-power interaction in efficacy, whereas in the viral model α scales drug modulation of biologically meaningful processes (e.g., infectivity β and production p ). Notably, α values were systematically smaller in the viral model than in the Bliss PD benchmark, consistent with the idea that mechanistic propagation through viral dynamics can reproduce observed interaction patterns without requiring large phenomenological nonlinearity. These findings complement prior synergy frameworks based on Bliss/Loewe principles (Chou, 2006; Chou, 2010; Jilek et al., 2012) and data-driven predictors (Shayan Majidifar et al., 2024; Wengong Jin et al., 2021; Kim et al., 2021; Ianevski et al., 2022; Pavel Sidorov et al., 2018) by showing that a mechanistic formulation can retain predictive accuracy while improving interpretability (Fitzgerald et al., 2006; Wagoner et al., 2022; Wein et al., 1998).

Practically, our results support a data-efficient strategy for prioritizing antiviral combinations when exhaustive screening is infeasible: the Bliss PD model offers a fast, computationally simple baseline, while the viral model provides improved synergy accuracy and mechanistic interpretability by explicitly linking drug actions to viral processes. This is particularly valuable for emerging infections such as SARS-CoV-2, where time and experimental capacity are limited (Gastine et al., 2021; Wagoner et al., 2022; Padmanabhan et al., 2020).

This study has limitations. The full efficacy and synergy landscapes were inferred from sparse diagonal measurements rather than directly observed across the full concentration grid, so predictions in unmeasured regions depend in part on model-based extrapolation. In addition, validation was performed within the same published dataset; therefore, further evaluation using independent datasets will be important to assess generalizability. Finally, analyses were based on *in vitro* Calu-3 data and a specific three-drug set, which may limit direct extension to other antiviral systems.

The kinetic parameters β,p,δ , and c represent important modeling assumptions in the standard target-cell infection model because they define the viral kinetic background through which drug effects are propagated. Accordingly, the fitted interaction parameter α is estimated within the viral kinetic regime specified by the selected parameter values. Consistent with this dependence, perturbing β,p,δ, or c in sensitivity analysis altered both the fitted α values and the prediction error. These results emphasize that appropriate virus-specific kinetic parameters should be used whenever possible when applying mechanistic viral-dynamics models to antiviral combination prediction.

Although α in the viral model is mechanistically placed, it remains a lumped interaction factor; future work should connect it more directly to molecular mechanisms and extend the model to include additional host processes, such as innate immune effects, and larger datasets. Hybrid approaches that combine mechanistic structure with data-driven components may further improve extrapolation under extreme sparsity (Shayan Majidifar et al., 2024; Kim et al., 2021).

In conclusion, our results suggest that efficacy and synergy landscapes can be approximated from sparse diagonal measurements, and that a mechanistic viral dynamics model can improve synergy prediction accuracy and interpretability compared with a Bliss PD benchmark. Together, these complementary frameworks provide a practical and biologically grounded toolkit for accelerating antiviral combination optimization under realistic experimental constraints.

This comparative approach is not limited to the specific antivirals tested here but serves as a robust and complementary strategy for evaluating drug combinations against other pathogens. Future work can leverage this framework to guide the experimental prioritization of novel drug candidates, strengthening the evaluation of antiviral therapies and accelerating the translation of effective combinations into clinical practice.

## Data Availability

The original contributions presented in the study are included in the article/supplementary material, further inquiries can be directed to the corresponding author.
